# Complement receptor 1 genetic polymorphism contributes to sporadic Alzheimer’s disease susceptibility in Caucasians: a meta-analysis

**DOI:** 10.1042/BSR20200321

**Published:** 2020-06-02

**Authors:** Hai Yuan, Lingling Du, Pingping Ge

**Affiliations:** Department of Rehabilitation Medicine, The Second People’s Hospital of Hefei City, Hefei, China

**Keywords:** Alzheimer's disease, complement receptor 1, gene, meta-analysis, polymorphism

## Abstract

Complement receptor 1 (CR1) plays an important role in the development of sporadic Alzheimer’s disease (SAD) in Caucasians. However, the influence of CR1 (rs6656401A/G and rs3818361T/C) genetic polymorphisms on the risk of SAD remains controversial. A meta-analysis of 18 case–control studies was performed to derive a more precise association of CR1 (rs6656401A/G or rs3818361T/C) genetic polymorphism with the risk of SAD in Caucasians. A statistical difference was found in the dominant model (odds ratio (OR): 1.23, 95% confidence interval (CI): 1.16–1.30, *P*=0.00), recessive model (OR: 1.28, 95% CI: 1.05–1.56, *P=*0.02), homozygote comparison (OR: 1.36, 95% CI: 1.12–1.66, *P=*0.002) or heterozygote comparison (AG versus GG) (OR: 1.21, 95% CI: 1.15–1.29, *P*=0.00) of CR1 rs6656401A/G. For CR1 rs3818361T/C, a statistical difference was observed in the dominant model (OR: 1.21, 95% CI: 1.13–1.31, *P=*0.00), recessive model (OR: 1.28, 95% CI: 1.07–1.53, *P*=0.006), homozygote comparison (OR: 1.35, 95% CI: 1.13–1.62, *P=*0.001) or heterozygote comparison (TC versus CC) (OR: 1.20, 95% CI: 1.11–1.29, *P=*0.00). In summary, despite some limitations, the present meta-analysis indicated that rs6656401A/G or rs3818361T/C polymorphism was related to SAD risk. Moreover, a carrier of rs6656401A/G or T carrier of rs3818361T/C in CR1 genetic polymorphism might be an increased factor for SAD in Caucasians.

## Introduction

Alzheimer’s disease (AD) is the most common neurodegenerative disease causing progressive memory impairment and cognitive dysfunction among elderly people [1,2]. The pathological hallmark of the disease is the accumulation of amyloid plaques in the brain, which leads to neurodegeneration [3]. Increasing evidence points to an important role of immunopathological processes in AD pathogenesis. Activated microglia and astrocytes produce strong immunopathological responses, which have been considered to contribute to AD neurodegeneration [4,5]. Although several research and clinical trials have shown that immunopathological responses are a key feature in AD brain [6], there is no effective treatment for this terminal disease.

Complement receptor 1 (CR1), located on chromosome 1q32, is a receptor for the complement component (3b/4b) [7,8], and is a member of the regulators of complement reactivation family that mediate immune responses. The extracellular portion of CR1 can be divided into 30 complement control protein repeats (CCPs), each comprising 59–75 amino acids [8,9]. The common isoform of CR1 as well as CR1*2 was found in ∼11% of Caucasians [10]. CR1 was postulated to be a key factor for AD pathogenesis due to its role in regulating complement activity by acting as a receptor of complement C3b protein [12]. Changes in CR1 expression levels in the CSF have been identified in the AD brain [13,14]. Moreover, CR1 was found to be associated with neuronal death in AD [11]. CR1-mediated phagocytosis is involved in the clearance of amyloid plaques and plays an important role in the AD neuropathology [15]. Aβ has been shown to activate the complement system by means of C1q, which binds to CR1 [16,17]. Crehan et al. found a possible association between increased CR1 and more active microglia, and the microglial ability to phagocytose Aβ was impeded through blocking CR1 [11]. Rogers et al. found that the CR1 protein was bound to Aβ42 peptide at its C3b ligation site, resulting in the clearance of Aβ [9], which may affect the Aβ42 peptide accumulation in AD [18]. Therefore, CR1 is important for the clearance of amyloid plaques, and is involved in the pathogenesis of AD.

However, some studies reported the vast majority of CR1 is detected in the peripheral erythrocytes and not in human brain [19–22]. And CR1 is associated with the pathophysiology of AD by mediating peripheral erythrocytes to capture circulating Aβ, and CR1 SNPs contribute to AD risk by altering erythrocyte CR1 expression [23]. Brouwers et al. showed that the CR1*2 isoform was expressed on the surface of erythrocytes of AD patients and was associated with AD risk [24]. Mahmoudi et al. found that rs6656401A/G and rs3818361T/C were strongly associated with the CR1*2 isoform at the protein and gene levels in AD patients [10]. Lambert et al. first found that CR1 rs6656401 A/G or rs3818361 T/C was associated with AD risk in Caucasians [25]. Several epidemiological studies were conducted to analyze the relationship between CR1 variants and AD susceptibility, although with inconsistent results [26–32]. Therefore, in the present study, a meta-analysis was conducted to assess the association between the CR1 SNP rs6656401A/G or rs3818361T/C and sporadic AD (SAD) risk in Caucasians in order to better understand the genetic mechanism of SAD before implementing efficient strategies for the prevention and management of this disease.

## Materials and methods

### Literature search

The Medline, Embase and HuGHESNet electronic databases were searched to identify all eligible articles before March 2019 that were conducted on human subjects, without language restriction. The combinations of the following Medical Subject Heading (MESH) terms and text words were adopted: (‘Alzheimer’s disease’ or ‘AD’) and (‘Complement receptor 1’ or ‘CR1’) and (‘polymorphism’ or ‘mutation’ or ‘genes’). The references of all relevant studies were also reviewed for additional relevant publications.

### Inclusion and exclusion criteria

The inclusion criteria were: (i) SAD was clinically diagnosed [33,34], (ii) case–control design and (iii) available genotypic distributions in cases and controls. The exclusion criteria were: (i) a family history of dementia, (ii) case reports, editorials and review articles, and (iii) unavailable data. Studies with more than one sample were considered as different comparisons.

### Data extraction

All studies were independently reviewed by two investigators (Lingling Du and Pingping Ge), and discrepancies were resolved by discussions. The following characteristics of eligible studies were extracted: first author, year of publication, country, genotyping method, ethnicity and clinical characteristics (age, gender etc). The quality evaluation score was calculated based on the Newcastle–Ottawa Scale (NOS) [35].

### Statistical analysis

The genotype distribution of the control population in eligible studies was tested for deviation from the Hardy–Weinberg equilibrium (HWE) using the chi-square test (with *P*≤0.1 considered as significant). Any study in which the genotype distribution was not in accordance with HWE was excluded.

The heterogeneity among the studies was evaluated with Cochran’s Q and the *I^2^* statistic (*P*>0.10 was considered representative of homogeneity). The significance of odds ratio (OR) and 95% confidence interval (CI) were determined based on the fixed-effect model (Mantel–Haenszel method) (*P_heterogeneity_*>0.10) [36]. Otherwise, the random-effects model (Der Simonian–Laird) was adopted using the STATA 12.0 or Review Manager 5.3 software [37]. Five different ORs were calculated in the present study for rs6656401A/G polymorphism: dominant model [(AA+AG) versus GG], recessive model [AA versus (AG+GG)], homozygote comparison (AA versus GG) and heterozygote comparison (AG versus GG, AA versus AG). The same method was applied to rs3818361T/C polymorphism. The statistical significance of the pooled ORs was determined by the Z-test and *P*≤0.05 was considered statistically significant.

The visual Begg’s funnel plot and the Egger’s linear regression test [38] were utilized to assess the publication bias with STATA 12.0 software (STATA Corp., College Station, TX, U.S.A.) (*P*≤0.10 was considered statistically significant). Individual studies were sequentially removed to explore the influence of each individual study on the pooled OR and the stability of the combined results.

### Study selection

**Figure 1:**
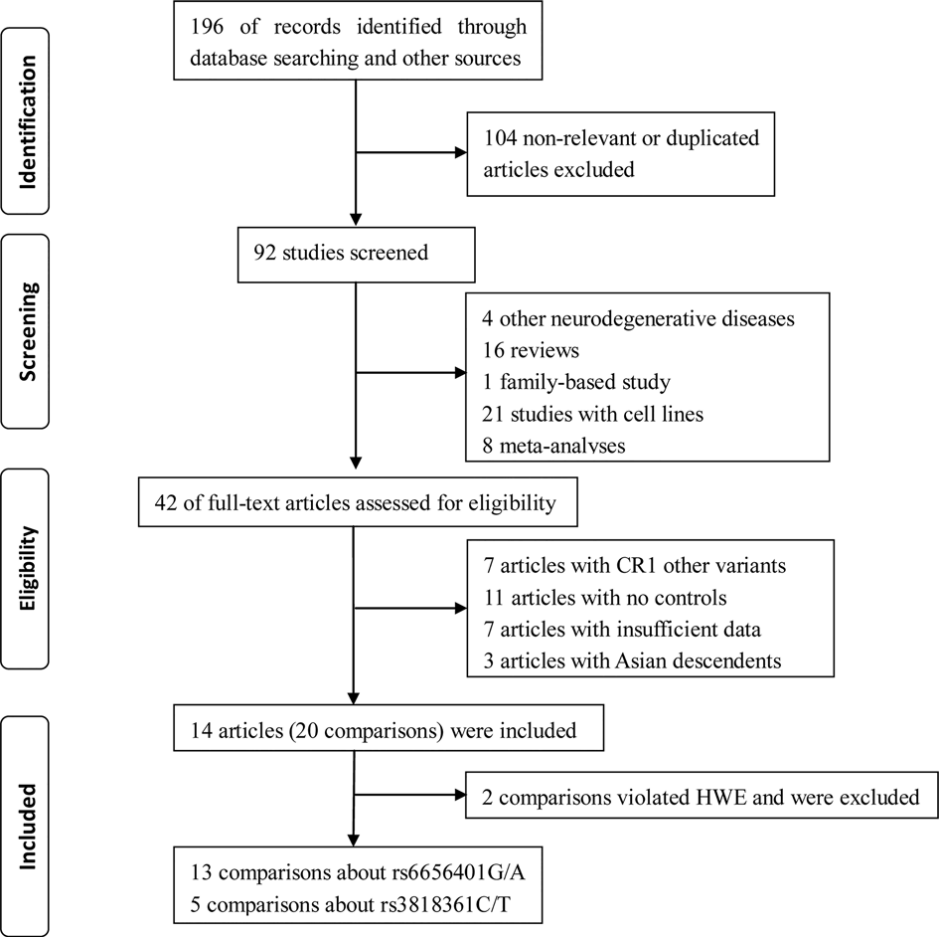
The PRISMA checklist of literature search and study selection

A total of 196 studies were identified based on a comprehensive search of databases and other sources. A total of 104 duplicated or non-relevant studies were removed after the primary screening. Based on further screening of the title or abstract, 50 studies were excluded (4 other neurodegenerative diseases, 16 reviews, 21 studies involving cell lines, 8 meta-analyses and 1 family-based study). After a detailed full-text review for eligibility, seven studies with other CR1 variants, seven articles with insufficient data, three studies of Asian descendants, and eleven studies with no controls were excluded. Finally, 12 studies pertaining to CR1 (rs6656401A/G and rs3818361T/C) polymorphisms and SAD risk were included [25–32,39–42]. In study selection, different comparisons were considered based on different district populations in two studies in the present meta-analysis [25,42]. Five comparisons were considered in one study [25]. Finally, 18 comparisons concerning CR1 rs6656401A/G (13 comparisons) or rs3818361T/C polymorphism (5 comparisons) were considered. The PRISMA checklist is shown in Figure 1.

## Results

### Study characteristics

There were 10704/12360 (rs6656401A/G) cases/controls or 4740/8495 (rs3818361T/C) cases/controls included in this meta-analysis. Non-dementia, age- and sex-matched controls were included in most studies [26–28,30–42,39–41]. Diagnoses of definite or probable SAD were established according to the National Institute of Neurological and Communicative Disorders and Stroke and the Alzheimer’s Disease and Related Disorders Association (NINCDS-ADRDA) [26–28,31,32,39,41]. Genomic DNA was extracted from blood according to standard procedure [26,28,32]. The real-time polymerase chain reaction-restriction (RT-PCR) or PCR-restriction fragment length polymorphism (PCR-RFLP) was performed to determine the genotypes in some studies [26–28,30,32,39]. The genetic distribution and frequencies of CR1 polymorphism among SAD cases and controls are exhibited in Table 1.

**Table 1:** Genetic distribution and frequencies for CR1 polymorphism among SAD cases and controls

Gene/Author	Genotypes						HWE		NOS score
	Cases			Controls			*χ*^2^	*P*	
	GG	AG	AA	GG	AG	AA			
rs6656401A/G									
Dos Santos	56	21	1	95	40	2	0.944	0.331	6
Van Cauwenberghe	635	378	39	319	130	20	2.044	0.153	7
Klimkowicz-Mrowiec	124	107	22	125	102	13	1.802	0.18	7
Toral-Rios	69	20	5	68	24	8	6.25	0.012	8
Hamilton	287	147	22	326	124	9	0.501	0.479	6
Omoumi	377	172	31	371	137	16	0.588	0.443	6
Santos-Rebouças	42	10	7	124	46	4	0.012	0.971	6
Kamboh	820	463	65	848	451	60	0	0.997	8
Corneveaux	634	339	45	402	171	18	0.001	0.972	7
Lambert (a)	1246	684	95	3601	1558	169	0.001	0.976	6
Lambert (b)	644	383	39	339	140	21	1.777	0.183	6
Lambert (c)	367	222	19	442	196	16	1.106	0.293	6
Lambert (d)	949	467	56	804	385	54	0.833	0.361	6
Lambert (e)	483	216	35	572	198	31	6.622	0.01	6
Li	431	227	31	458	197	27	0.992	0.319	6

Gene/Author	Genotypes						HWE		NOS score
	Cases			Controls			*χ*^2^	*P*	
	CC	TC	TT	CC	TC	TT			
rs3818361T/C									
Hamilton	278	152	21	305	124	10	0.396	0.529	6
Omoumi	375	170	35	364	141	19	1.312	0.252	6
Harold (a)	1427	712	87	3312	1367	157	1.196	0.274	9
Harold (b)	337	182	35	524	260	40	1.099	0.295	9
Harold (c)	706	401	52	1420	678	90	0.637	0.425	9

### Meta-analysis

**Figure 2:**
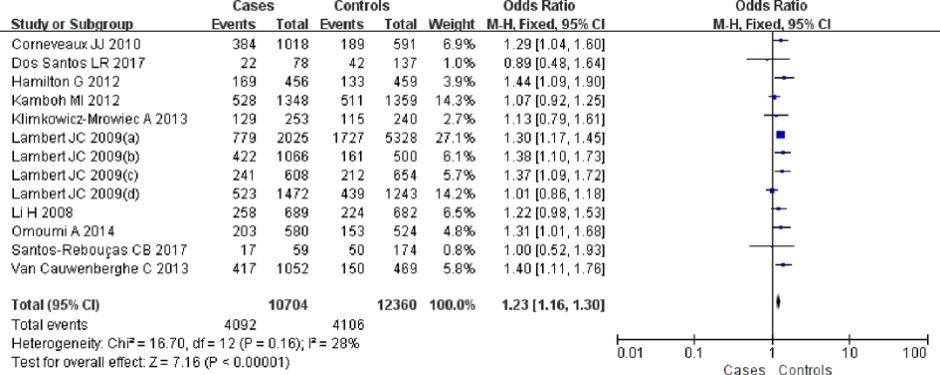
Forest plot for rs6656401A/G genetic polymorphism ((AA+AG) versus GG) and SAD susceptibility

**Figure 3:**
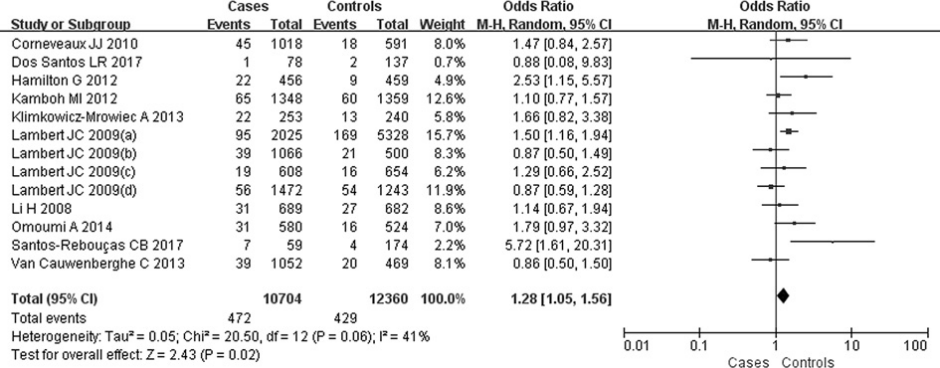
Forest plot for rs6656401A/G genetic polymorphism (AA versus (AG+GG)) and SAD susceptibility

**Figure 4:**
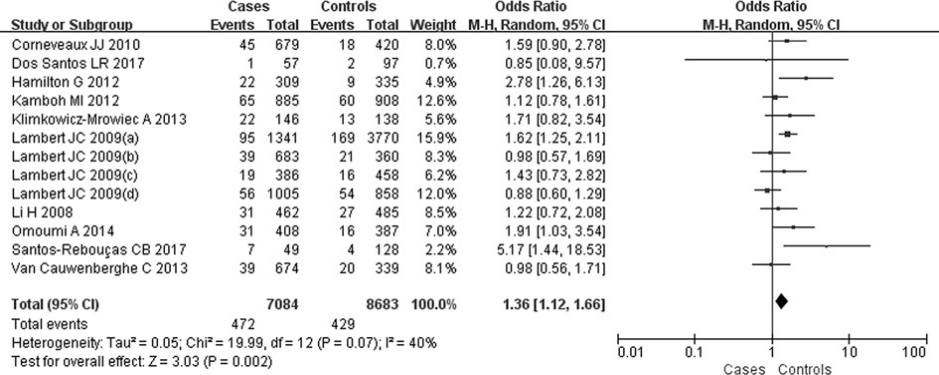
Forest plot for rs6656401A/G genetic polymorphism (AA versus GG) and SAD susceptibility

**Figure 5:**
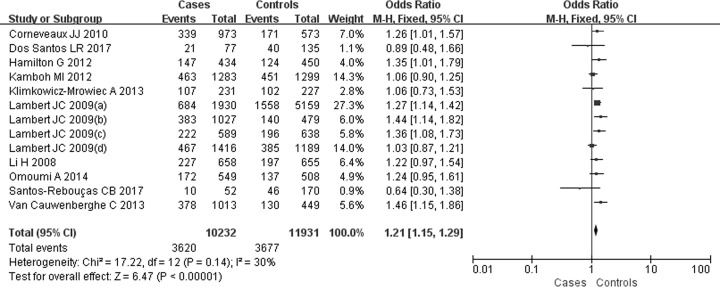
Forest plot for rs6656401A/G genetic polymorphism (AG versus GG) and SAD susceptibility

For the SNP rs6656401A/G, 13 comparisons were analyzed in Caucasian populations [25–32,39–41]. A significantly increased SAD risk was observed for A carriers in ((AA+AG) versus GG: OR = 1.23, 95% CI: 1.16–1.30, *P*=0.00 (Figure 2)), (AA versus (AG+GG): OR = 1.28, 95% CI: 1.05–1.56, *P=*0.02 (Figure 3)), (AA versus GG: OR = 1.36, 95% CI: 1.12–1.66, *P=*0.002) (Figure 4) or (AG versus GG: OR = 1.21, 95% CI: 1.15–1.29, *P=*0.00) (Figure 5). These implied that A carrier might be an increased factor for SAD risk. The summary results are presented in Table 2.

**Table 2:** The results of meta-analysis in overalls

Gene	Gene polymorphism	Test of heterogeneity			Analysis model	Test of association		
		*χ^*2*^*	*P*	*I^2^*		*OR*	*95% CI*	*P*
rs6656401G/A	AA vs AG+GG	20.5	0.06	41	R	1.28	1.05, 1.56	0.02
	AA+AG vs GG	16.7	0.16	28	F	1.23	1.16, 1.30	0.00
	AG vs GG	17.22	0.14	30	F	1.21	1.15, 1.29	0.00
	AA vs AG	20.93	0.05	43	R	1.13	0.91, 1.39	0.26
	AA vs GG	19.99	0.07	40	R	1.36	1.12, 1.66	0.002
rs3818361C/T	TT vs TC+CC	3.51	0.48	0	F	1.28	1.07, 1.53	0.006
	TT+TC vs CC	1.77	0.78	0	F	1.21	1.13, 1.31	0.00
	TC vs CC	1.33	0.86	0	F	1.20	1.11, 1.29	0.00
	TT vs TC	3.00	0.56	0	F	1.13	0.94, 1.36	0.18
	TT vs CC	3.58	0.47	0	F	1.35	1.13, 1.62	0.001

Abbreviations: F, fixed-effect; R, random-effect.

Five comparisons were conducted for the SNP rs3818361T/C [30,31,42]. A higher frequency of T carriers in SAD risk was revealed in ((TT+TC) versus CC: OR = 1.21, 95% CI: 1.13–1.31, *P*=0.00, TT versus (TC+CC): OR = 1.28, 95% CI: 1.07–1.53, *P*=0.006, TT versus CC: OR = 1.35, 95% CI: 1.13–1.62, *P*=0.001 or TC versus CC: OR = 1.20, 95% CI: 1.11–1.29, *P*=0.00). So, T carrier of rs3818361T/C might be an increased factor for SAD risk (Table 2).

### Sensitivity and publication bias

**Figure 6:**
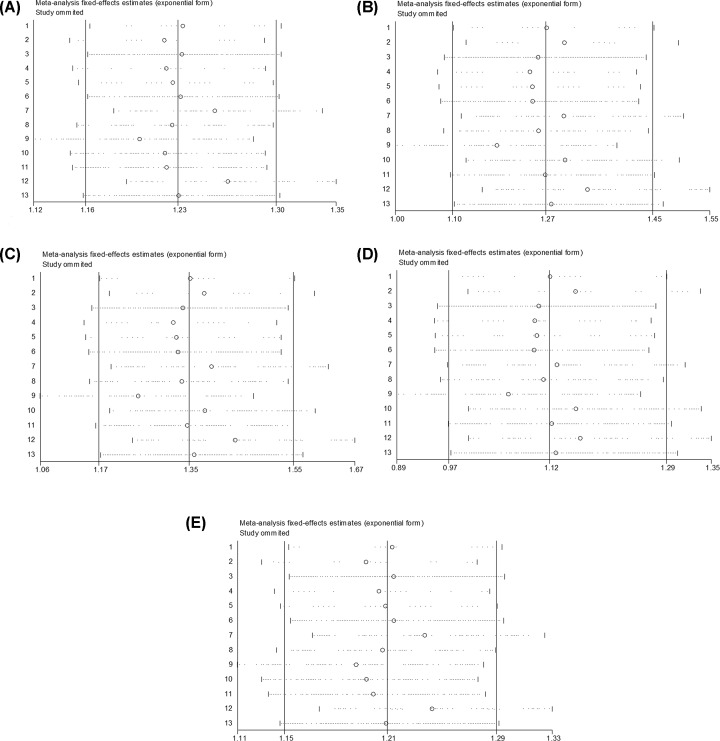
Sensitivity analysis for the relation of rs6656401A/G genetic polymorphism with SAD susceptibility (**A**) (AA+AG) versus GG, (**B**) AA versus (AG+GG), (**C**) AA versus GG, (**D**) AA versus AG, (**E**) AG versus GG.

**Figure 7:**
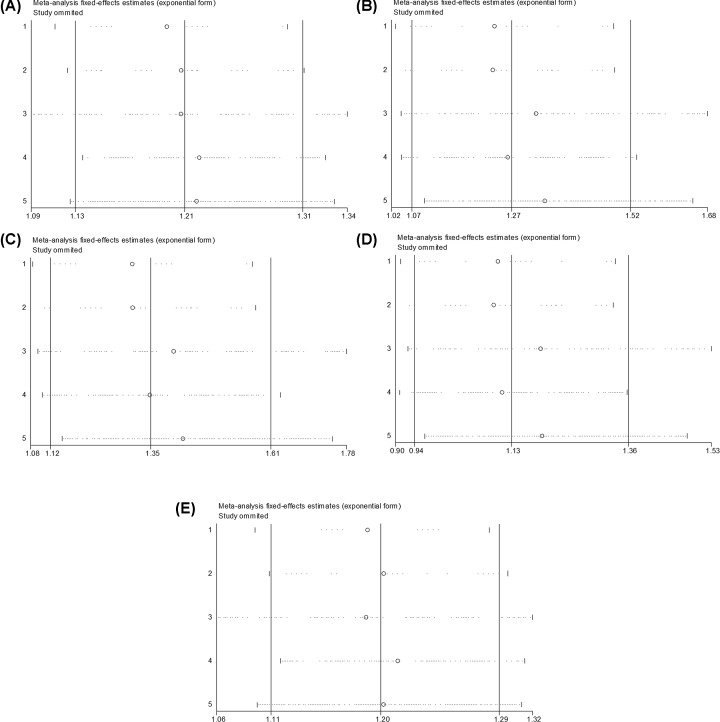
Sensitivity analysis for the relation of rs3818361T/C genetic polymorphism with SAD susceptibility (**A**) (TT+TC) versus CC, (**B**) TT versus (TC+CC), (**C**) TT versus CC, (**D**) TT versus TC, (**E**) TC versus CC.

The sensitivity analyses were performed by sequential removal of individual studies to evaluate the effect on the overall ORs for rs6656401A/G ((AA+AG) versus GG, AA versus (AG+GG), AA versus GG, AA versus AG and AG versus GG) (Figure 6A–E) and rs3818361T/C ((TT+TC) versus CC, TT versus (TC+CC), TT versus CC, TT versus TC and TC versus CC) (Figure 7A–E). No study affected the pooled results in the above three models, indicating that the present study results were relatively reliable and stable.

**Figure 8:**
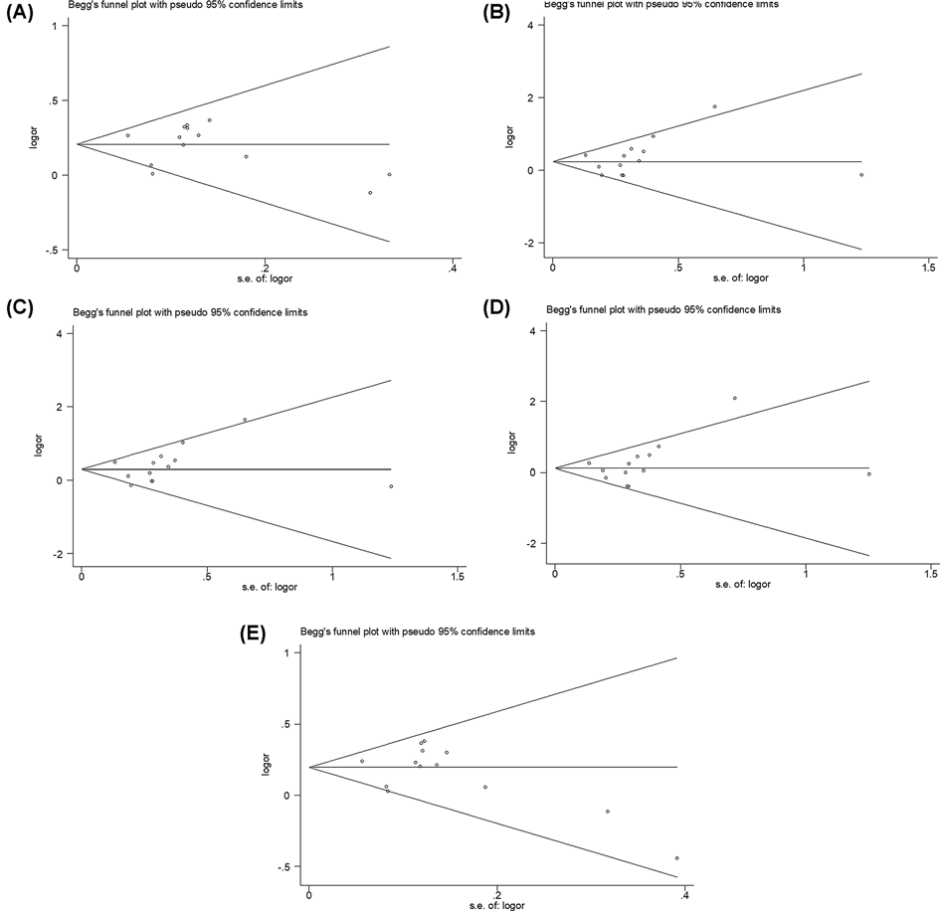
Begg’s funnel plot for analysis models in CR1 rs6656401A/G genetic polymorphism (**A**) (AA+AG) versus GG, (**B**) AA versus (AG+GG), (**C**) AA versus GG, (**D**) AA versus AG, (**E**) AG versus GG.

**Figure 9:**
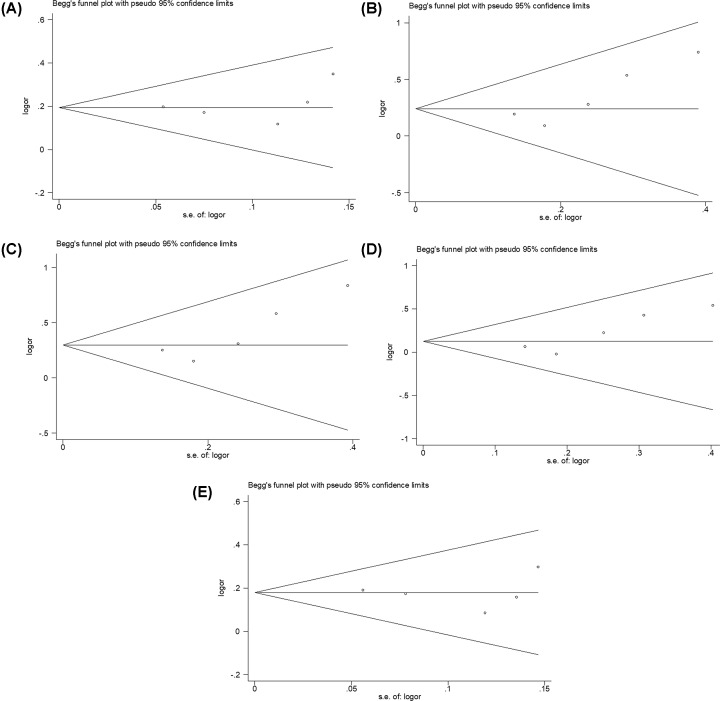
Begg’s funnel plot for analysis models in CR1 rs3818361T/C genetic polymorphism (**A**) (TT+TC) versus CC, (**B**) TT versus (TC+CC), (**C**) TT versus CC, (**D**) TT versus TC, (**E**) TC versus CC.

The shape of the Begg’s funnel plots in genetic models seemed nearly symmetrical, indicating that no evidences for obvious publication bias were exhibited (Figures 8A–E and 9A–E). Based on Egger’s linear regression test, no significant publication bias were also determined in rs6656401A/G genetic models ((AA+AG) versus GG, t = −0.16, *P*=0.875; AA versus (AG+GG), t = 0.84, *P*=0.417; AA versus GG, t = 0.80, *P*=0.442; AA versus AG, t = 0.92, *P*=0.379; and AG versus GG, t = −0.61, *P*=0.557) and rs3818361T/C genetic models ((TT+TC) versus CC, t = 0.59, *P*=0.597 and TC versus CC, t = −0.08, *P*=0.943). However, a statistic difference was found in rs3818361T/C genetic model (TT versus (TC+CC), t = 2.94, *P*=0.061; TT versus CC, t = 2.77, *P*=0.069; and TT versus TC, t = 3.34, *P*=0.044).

## Discussion

Longer alleles of CR1 were found to be risk factors for the development of AD, based on the excessive inhibition of C3b or C4b and the decrease in C3b-mediated opsonization of the amyloid b42 (Ab42) peptide [10,24,43]. The alterations in CR1 structure and expression caused by genetic variability could lead to an alteration of Ab42 clearance [9,44]. Clinically, CR1 variants were also associated with neuroimaging features of AD [45] and neuritic plaque burden in AD brains [46]. In addition, the CR1 locus (rs6656401A/G) had an important effect on global cognitive dysfunction due to the enhanced burden of AD-related neuropathology, such as the deposition of amyloid plaques [47–49]. Therefore, CR1 is considered as a biological candidate gene for the development of AD.

In a genome-wide association study, Lambert et al. found the SNPs of CR1, rs6656401A/G and rs3818361T/C, in AD patients [25]. Another study found that the SNP rs6656401A/G was associated with AD risk, and rs3818361 T/C was related to AD risk in APOEε4 carriers [50]. Chibnik et al. found a correlation of the A allele r6656401A/G CR1 with deposition of neuritic plaques [49]. Genotype rs6656401A/G was also reported to be associated with severity of CAA pathology at autopsy (OR = 1.34, 95% CI: 1.05–1.71, *P*<0.009) [51]. An association of rs3818361T/C with a low amyloid burden was observed in the brain of AD patients, which emphasized the potential implication of CR1 in the brain amyloid pathway [52]. In the present study, CR1 rs6656401A/G or rs3818361T/C polymorphisms were identified as risk factors for SAD, indicating that individuals with A carrier of rs6656401A/G or T carrier of rs3818361T/C might be at higher risk of SAD. This meta-analysis supported the hypothesis of most previous studies that CR1 rs6656401A/G or rs3818361T/C polymorphism was associated with the risk of SAD. Lambert et al. found that CR1 SNP rs6656401A/G was a risk factor for AD susceptibility in a Caucasian population (OR = 1.21, 95% CI: 1.14–1.29, *P*=3.7 × 10^−9^ for combined data) [25].

A prevalence study between 428 AD cases and 524 controls implicated a significant association of rs6656401A/G or rs3818361T/C with AD risk [31]. Keenan et al. also suggested a strong linkage disequilibrium between SNP rs6656401A/G and AD risk (*P*=0.012) [53]. Other studies confirmed the same result, as well as a significant association of rs6656401G/A or rs3818361T/C of CR1 with AD risk [29,27,31,54]. However, the evidence of high heterogeneity was found in some models [AA versus (AG+GG), AA versus AG and AA versus GG]. In inclusive studies, Dos Santos et al. demonstrated no association between AD and rs6656401A/G CR1 in 79 AD patients and 145 healthy controls in a Brazilian population, which might play a role in the contradictory results [26]. These findings were also replicated in the distribution of the rs6656401 A/G of CR1 by Klimkowicz-Mrowiec et al. [28] and Santos-Rebouças et al. [32]. Klimkowicz-Mrowiec et al. found that the genetic interaction with the APOE ε4 carriers might be related to the risk of AD [28]. In addition, Hamilton et al. found that gender played a critical role in genetic risk of AD [30]. Hence, the relevant subgroup analysis should have been conducted based on age at onset, gender and ethnicity to evaluate if heterogeneity influenced the results of the meta-analysis. However, most studies did not report original and adequate information, which made it difficult to conduct further analysis. In the present study, the results of the Begg’s funnel plot and the Egger’s regression test reduced the potential for publication bias. The results of *I*^2^ (*41, 43* and *40%*, respectively) showed that the proportion of interstudy variability contributed to low heterogeneity. In addition, the results of sensitivity analysis based on the sequential removal of individual studies showed that no study had any effect on the pooled results in the above three models. So, the pooled results of the above three models were stable and credible.

The current meta-analysis had some limitations. First, the sample size of included studies was small, which might contribute to possible limited strength of the statistics. However, the latest high quality studies (NOS score > 5), which met our stringent selection criteria, were included in the meta-analysis. Second, SAD has complex etiopathogenesis, and the gene–gene and gene–environment relationships were not analyzed due to lack of original data. Larger sample studies with multifactorial etiology should be conducted in the future. Finally, a possible publication bias might be explored by the results of the Egger’s linear regression test in rs3818361T/C genetic models (TT versus (TC+CC), TT versus CC and TT versus TC), it is possible that relevant unpublished articles with null results were not included. Although the shape of the visual Begg’s funnel plot appeared to be approximately symmetrical and the results of sensitivity analysis suggest these analysis models are stable and reliable, the results of rs3818361T/C should be applied with caution.

Despite these limitations, the current meta-analysis suggested that the CR1 rs6656401 A/G or rs3818361T/C polymorphism might be a risk factor for SAD. The rs6656401 A/G or rs3818361T/C genetic polymorphism plays an important role in the development of SAD. A carrier of rs6656401A/G or T carrier of rs3818361T/C CR1 genetic polymorphism might be an increased factor for SAD.

## Abbreviations

Ab42: amyloid b42
AD: Alzheimer’s disease
APOE: apolipoprotein E
Aβ: β-Amyloid protein
CI: confidence interval
CR1: complement receptor 1
CSF: cerebrospinal fluid
HWE: Hardy–Weinberg equilibrium
NOS: Newcastle–Ottawa Scale
OR: odds ratio
SAD: sporadic AD
SNP: single nucleotide polymorphism
